# An Anonymous User Authentication with Key Agreement Scheme without Pairings for Multiserver Architecture Using SCPKs

**DOI:** 10.1155/2013/419592

**Published:** 2013-06-09

**Authors:** Peng Jiang, Qiaoyan Wen, Wenmin Li, Zhengping Jin, Hua Zhang

**Affiliations:** State Key Laboratory of Networking and Switching Technology, Beijing University of Posts and Telecommunications, Beijing 100876, China

## Abstract

With advancement of computer community and widespread dissemination of network applications, users generally need multiple servers to provide different services. Accordingly, the multiserver architecture has been prevalent, and designing a secure and efficient remote user authentication under multiserver architecture becomes a nontrivial challenge. In last decade, various remote user authentication protocols have been put forward to correspond to the multi-server scenario requirements. However, these schemes suffered from certain security problems or their cost consumption exceeded users' own constrained ability. In this paper, we present an anonymous remote user authentication with key agreement scheme for multi-server architecture employing self-certified public keys without pairings. The proposed scheme can not only retain previous schemes' advantages but also achieve user privacy concern. Moreover, our proposal can gain higher efficiency by removing the pairings operation compared with the related schemes. Through analysis and comparison with the related schemes, we can say that our proposal is in accordance with the scenario requirements and feasible to the multi-server architecture.

## 1. Introduction

 In modern society, people's life is highly dependent on the Internet, but the exposure of networks often causes great loss to users, which brings about that a secure user authentication mechanism has become the key issue to preserve valid remote clients in safety from being attacked. There is no doubt that the user authentication with smart card is one of the most widely used and the simplest approaches. When taking only one sort of service into account, some password authentication schemes for single-server environment have been proposed [1, 2].

Later with the rapid development of technology, different servers are needed to offer service via the network, and conventional methods need users to register with various servers repetitively and remember different identities and passwords. It is obvious that these traditional schemes make authentication inconvenient and cost much. Consequently, an appropriate multiserver user authentication mechanism has turned into a concern. In 2001, Li et al. [3] gave a remote user authentication scheme in neural networks for the first time, which opened up the gateway access to the multiserver architecture.

Considering the system environment without loss of generality, the multiserver architecture consists of multiple distributed service servers and remote clients with limited resource and capability. The service servers offer different access services such as e-commerce, online conference, network game, and remote medical system. If a remote client wants to access to these services, he/she needs to login these service servers through cellular network or wireless local area networks (WLANs).

Due to multiserver environment special characteristics and information security problem in public networks, designing a feasible user authentication scheme under multiserver architecture is a key issue, which can ensure the access of legitimate users and prevent invalid user from interfering with the service server. A practical user authentication scheme under the multiserver environment must address the following requirements. They consist of both the previous criteria [1] and new user anonymity issue.

1. No repetitive registration is needed for the multiserver environments.
2. No verification table is stored in the server.
3. Mutual authentication and session key agreement can be achieved between the users and the service servers to carry on subsequent communications.
4. Various possible attacks can be resisted.
5. User can choose identity and password freely and change his/her password freely.
6. The computational and communication cost is low since the energy resources and computing capability of a smart card are limited.
7. The user is not allowed to expose his identity privacy information to eavesdroppers. Assume that the adversary obtains a valid user's identity, he/she can masquerade the user to enjoy the regular service without registration, which can cause losses for the valid user or even worse consequences. So the anonymous authentication should be implemented.

In order to satisfy all of these criteria, this paper proposes an anonymous remote user authentication scheme without pairings for multiserver architecture using self-certified public keys (SCPKs). We present public key-based user anonymous authentication scheme under the multiserver environment. Meanwhile, our proposal heightens efficiency increasingly accompanied by the removal of pairings operation; in contrast, the existing public key-based authentication schemes generally employ pairings function. Moreover, our proposal can avoid the server spoofing attack since the verification process relies on the server's private key. Through security and performance analysis, our proposal not only achieves anonymous authentication with key agreement securely but also results more efficiently, remedying the weaknesses of previous authentication schemes which either encounter some attacks or fail to protect user privacy or cost relatively more energy. Compared with other related achievements, ours is more suitable for the remote user whose resources and capability are constrained under multiserver architecture.

The rest of this paper is organized as follows.  Section 2 briefly describes some related works. Some preliminaries are given in Section 3. Our proposed secure and efficient user authentication scheme for multiserver architecture and corresponding analysis are presented in Sections 4 and 5, respectively. Finally, some conclusions are drawn in Section 6.

## 2. Related Work

 Until now, two categories of improved multiserver user authentication schemes, hash-based authentication and public key based authentication, have emerged successively. To hash-based authentication, some user password authentication suggestions [4–7] based on static ID have been proposed to conquer the weaknesses of Li et al.'s, yet these were proven easy to be traced. In 2009, Liao and Wang [8] raised a dynamic identity authentication protocol for multiserver environment to advance previous work. In the following years, many researchers [9–12] have developed and enhanced the user authentication scheme step by step. To public key-based authentication, employing public key cryptosystem into the password authentication, Das et al. [13] first proposed a remote user authentication protocol with smart card using bilinear pairings. Yet theirs had an obvious disadvantage: no mutual authentication and key agreement. To improve the security, a series of user authentication schemes [14–16] with bilinear pairings have been presented. To improve the efficiency, Tseng et al. [17] gave a low-cost pairing-based user authentication protocol for wireless users and claimed that theirs was efficient, easy password changing, and suitable for multiserver environment in distributed networks. Unfortunately, in 2013, Liao and Hsiao [18] pointed out that Tseng et al.'s scheme also lacked mutual authentication with session key agreement, suffered from insider attack, password guessing attack, and replay attack, and advanced a pairings-based user authentication scheme using self-certified public keys. Liao and Hsiao claimed that their proposal could withstand various possible attacks and was well suited for multiserver environment.

Regretfully, most of the existing related public key based authentication schemes under multiserver architecture mentioned previously did not pay attention to user anonymity issue. Moreover, their authentication schemes needed excessive energy consumption employing pairings operation and suffered from the server spoofing attack, which was not conducive to communication running and trapped in DoS attack easily.

## 3. Preliminaries

 We now briefly review some basic concepts used in this paper, including bilinear pairings [19], related complexity assumptions [20], and self-certified public keys [21, 22].

### 3.1. Admissible Bilinear Pairing

Let *𝔾* be an additive group generated by *P* with prime order *q* and let *𝔾*_*T*_ be a multiplicative group of the same order. A map $\hat{e}:𝔾\times 𝔾\to {𝔾}_{T}$ is said to be an admissible bilinear pairing if the following three conditions hold true.

1. Bilinearity: for all *a*, *b* ∈ *ℤ*_q_*, we have $\hat{e}(aP,bP)=\hat{e}{\left(P,P\right)}^{ab}$.
2. Nondegeneracy: $\hat{e}(P,P)\neq 1_{{𝔾}_{T}}$.
3. Computability: $\hat{e}$ is efficiently computable.

We refer readers to [19] for more details of such pairings.

### 3.2. Complexity Assumption

1. Computational discrete logarithm (CDL) assumption: given *Q* = *k* · *P*, where *P*, *Q* ∈ *𝔾*, there exists no probabilistic polynomial-time algorithm which can determine *k*.
2. Computational Diffie-Hellman (CDH) assumption: given two elements *aP*, *bP* in a group *𝔾*, where the unknown numbers *a*, *b* ∈ *ℤ*_q_* are selected at random, there exists no probabilistic polynomial-time algorithm which can compute *a* *bP*.
3. Elliptic curve factorization (ECF) assumption: given two elements *P*, *Q*, where *Q* = *aP* + *bP* and *a*, *b* ∈ *ℤ*_q_*, there exists no probabilistic polynomial-time algorithm which can obtain *aP* and *bP*.

### 3.3. Self-Certified Public Key

Here, we describe a self-certified public key process briefly; more details can be found in [21, 22].

1. Initialization: given a group *𝔾* on an elliptic curve *E*, *P* is a based point generator of prime order *q*, the system authority (SA) selects a random value *s* ∈ *ℤ*_q_* as its private key and computes the public key *P*_pub_ = *s* · *P*. Publish the related parameters and keep *s* secret.
2. Partial private key and private key generation: the user *U*_*i*_ chooses a number *k*_*i*_ randomly, computes *K*_*i*_ = *k*_*i*_ · *P*, and sends (ID_*i*_, *K*_*i*_) to SA over a secure channel. SA calculates *W*_*i*_ = *K*_*i*_ + *w*_*i*_ · *P* as the witness using a random number *w*_*i*_. Then, SA computes the user's partial private key ${\overset{-}{s}}_{i}=H(\text{I}{\text{D}}_{i}||W_{i})\cdot s+w_{i}$ and submits $\left({\overset{-}{s}}_{i},W_{i}\right)$ to *U*_*i*_. *U*_*i*_ can obtain its private key $s_{i}={\overset{-}{s}}_{i}+k_{i}$.
3. Public key extraction: *U*_*i*_'s public key can be computed by Pub_*i*_ = *s*_*i*_ · *P*. Any entity, who communicates with *U*_*i*_ and receives the witness *W*_*i*_, can authenticate *U*_*i*_'s public key Pub_*i*_ as long as he/she calculates the equation: Pub_*i*_ = *H*(ID_*i*_||*W*_*i*_) · *P*_pub_ + *W*_*i*_.

## 4. The Proposed Scheme

**Figure 1:**
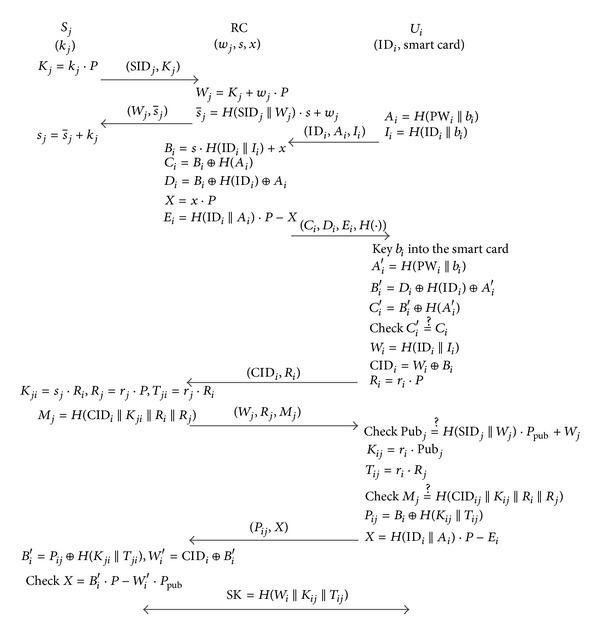
The proposed scheme.

 In this section, we propose an anonymous remote user authentication scheme for multiserver environment without pairings, which consists of five phases: server registration phase, user registration phase, login phase, verification phase, and password change phase. Three entities are involved: user (*U*_*i*_), service server (*S*_*j*_), and registration center (RC). RC chooses the system private/public key pair *s*/*P*_pub_, where *s* is a random number in *ℤ*_q_* and *P*_pub_ = *s* · *P*. Then publish the system parameters Params = {*𝔾*, *P*, *q*, *P*_pub_, *H*(·)} and keep *s* secret. The notations used in this section are listed in Table 1. Some detailed steps will be described as follows and shown in Figure 1.

### 4.1. Server Registration Phase

When the service server wants to access to the multiserver architecture, it needs to register first. In this phase, RC uses the self-certified public key (SCPK) to generate the related credentials.

*Step S1. S*_*j*_ chooses a random value *k*_*j*_ ∈ *ℤ*_q_*, computes *K*_*j*_ = *k*_*j*_ · *P*, and sends (SID_*j*_, *K*_*j*_) to RC.

*Step S2.* After receiving the message (SID_*j*_, *K*_*j*_), RC generates a *w*_*j*_ ∈ *ℤ*_q_* randomly, calculates *W*_*j*_ = *K*_*j*_ + *w*_*j*_ · *P*, ${\overset{-}{s}}_{j}=H({\text{SID}}_{j}||W_{j})\cdot s+w_{j}$, and issues $\left(W_{j},{\overset{-}{s}}_{j}\right)$ to *S*_*j*_.

*Step S3. S*_*j*_ can obtain its private key with $s_{j}={\overset{-}{s}}_{j}+k_{j}$ and verify the validity of the message by computing Pub_*j*_ = *s*_*j*_ · *P* = *H*(SID_*j*_||*W*_*j*_) · *P*_pub_ + *W*_*j*_.

If the equation holds, the issued values are valid, and vice versa.

### 4.2. User Registration Phase

Supposing that the user *U*_*i*_ wants to get service granted only from *S*_*j*_, he/she needs to register to the same RC that *S*_*j*_ did, by submitting his identity ID_*i*_ and password PW_*i*_ to RC. Then, RC returns the smart card back to *U*_*i*_. The communication between *U*_*i*_ and RC is through a secure channel. The steps are performed as follows.

*Step U1. U*_*i*_ freely chooses a password PW_*i*_ and a random number *b*_*i*_ to compute *A*_*i*_ = *H*(PW_*i*_||*b*_*i*_) and *I*_*i*_ = *H*(ID_*i*_||*b*_*i*_). Then, *U*_*i*_ submits (ID_*i*_, *A*_*i*_, *I*_*i*_) to RC for user registration via a secure channel.

*Step U2. *RC calculates

 
*B*_*i*_ = *s* · *H*(ID_*i*_||*I*_*i*_) + *x*,
 
*C*_*i*_ = *B*_*i*_ ⊕ *H*(*A*_*i*_),
 
*D*_*i*_ = *B*_*i*_ ⊕ *H*(ID_*i*_) ⊕ *A*_*i*_,
 
*X* = *x* · *P*,
 
*E*_*i*_ = *H*(ID_*i*_||*A*_*i*_) · *P* − *X*,

stores (*C*_*i*_, *D*_*i*_, *E*_*i*_, *H*(·)) in *U*_*i*_'s smart card, and submits it to *U*_*i*_. Then *U*_*i*_ keys *b*_*i*_ into the smart card.

### 4.3. Login Phase

 When *U*_*i*_ wants to login to the server *S*_*j*_, he/she first inserts his/her own smart card to a card reader and then inputs the identity ID_*i*_ and password PW_*i*_. The log-in details with respect to this smart card are as follows.

*Step L1.* The smart card computes *A*_*i*_′ = *H*(PW_*i*_||*b*_*i*_), *B*_*i*_′ = *D*_*i*_ ⊕ *H*(ID_*i*_) ⊕ *A*_*i*_′, and *C*_*i*_′ = *B*_*i*_′ ⊕ *H*(*A*_*i*_′) and checks whether *C*_*i*_′ = *C*_*i*_. If the answer is yes, it means that the smart card matches to *U*_*i*_.

*Step L2.* The smart card generates a random value *r*_*i*_ ∈ *ℤ*_q_* and computes

 
*W*_*i*_ = *H*(ID_*i*_||*I*_*i*_),
 CID_*i*_ = *W*_*i*_ ⊕ *B*_*i*_,
 
*R*_*i*_ = *r*_*i*_ · *P*.

*Step L3.* The smart card submits the login request message (CID_*i*_, *R*_*i*_) to *S*_*j*_ over a public channel.

### 4.4. Verification Phase

 After receiving the login request message from *U*_*i*_, *S*_*j*_ performs the following tasks to authenticate the user.

*Step V1. S*_*j*_ checks whether CID_*i*_ conforms to the fixed format. If the format is wrong, *S*_*j*_ outputs the reject message; otherwise it calculates

 
*K*_*ji*_ = *s*_*j*_ · *R*_*i*_,
 
*R*_*j*_ = *r*_*j*_ · *P*,
 
*T*_*ji*_ = *r*_*j*_ · *R*_*i*_,
 
*M*_*j*_ = *H*(CID_*i*_||*K*_*ji*_||*R*_*i*_||*R*_*j*_),

where *r*_*j*_ is a random value, chosen by *S*_*j*_. Then *S*_*j*_ sends (SID_*j*_, *W*_*j*_, *R*_*j*_, *M*_*j*_) to *U*_*i*_.

*Step V2.* Receiving the message (*W*_*j*_, *R*_*j*_, *M*_*j*_), *U*_*i*_ first verifies the public key of *S*_*j*_ by the equation Pub_*j*_ = *H*(SID_*j*_||*W*_*j*_) · *P*_pub_ + *W*_*j*_. Only under the case the equation holds, *U*_*i*_ continues to calculate *K*_*ij*_ = *r*_*i*_ · Pub_*j*_, *T*_*ij*_ = *r*_*i*_ · *R*_*j*_. Then *U*_*i*_ needs to check whether $M_{j}\overset{?}{=}H({\text{CID}}_{ij}||K_{ij}||R_{i}||R_{j})$. When the verification can pass, *U*_*i*_ authenticates *S*_*j*_ and computes

 
*P*_*ij*_ = *B*_*i*_ ⊕ *H*(*K*_*ij*_||*T*_*ij*_),
 
*X* = *H*(ID_*i*_||*A*_*i*_) · *P* − *E*_*i*_.

Then *U*_*i*_ transmits (*P*_*ij*_, *X*) to *S*_*j*_.

*Step V3.* Next, *S*_*j*_ undoes *B*_*i*_′ = *P*_*ij*_ ⊕ *H*(*K*_*ji*_||*T*_*ji*_), *W*_*i*_′ = CID_*i*_ ⊕ *B*_*i*_′ and examines $X\overset{?}{=}B_{i}^{\prime }\cdot P-W_{i}^{\prime }\cdot P_{\text{pub}}$. If it is not the case, *S*_*j*_ rejects the message and stops the session. Otherwise, *S*_*j*_ successfully authenticates *U*_*i*_.

*Step V4.* Finally, the user *U*_*i*_ and the service server *S*_*j*_ agree on a common session key as

 SK = *H*(*W*_*i*_||*K*_*ij*_||*T*_*ij*_).

### 4.5. Password Change Phase

 The password change phase is invoked when the user wants to change his/her password PW_*i*_ to a new password PW_i_*. The user first inserts his/her smart card into a card reader and enters ID_*i*_, PW_*i*_. The smart card computes *A*_*i*_ = *H*(PW_*i*_||*b*_*i*_), *B*_*i*_′ = *D*_*i*_ ⊕ *H*(ID_*i*_) ⊕ *A*_*i*_ and *C*_*i*_′ = *B*_*i*_′ ⊕ *H*(*A*_*i*_). Then, the smart card checks if the *C*_*i*_′ is the same as *C*_*i*_. If both values are the same, the user is asked to input a new password PW_i_*. The smart card calculates new information *A*_i_* = *H*(PW_i_*||*b*_*i*_), *C*_i_* = *C*_*i*_ ⊕ *H*(*A*_*i*_) ⊕ *H*(*A*_i_*), *D*_i_* = *D*_*i*_ ⊕ *A*_*i*_ ⊕ *A*_i_*, *E*_i_* = *H*(ID_*i*_||*A*_i_*) · *P* + *E*_*i*_ − *H*(ID_*i*_||*A*_*i*_) · *P*. At last, the smart card replaces *C*_*i*_, *D*_*i*_, *E*_*i*_ with the new *C*_i_*, *D*_i_*, *E*_i_* to accomplish changing password. In this phase, RC is not needed to participate and the user can freely complete changing password by himself.

## 5. Analysis of Our Scheme

 In this section, we first analyze the functionality features of our proposed scheme based on the requirements of the remote user authentication for multiserver architecture, which have been presented in Section 1. Then we evaluate the performance of the proposed scheme and make comparisons with some related works [8, 9, 11, 12, 17, 18].

### 5.1. No Repetitive Registration

 In our scheme, before the user wants to login to the server under multiserver environment, they must run the user registration with his/her information to the registration center. Then, the user can access to all the service without submitting registration request once again.

### 5.2. No Verification Table

Throughout the protocol process, it is not difficult to find that RC and *S*_*j*_ have no need to maintain any verification or password table, which can cost much and whose leakage may cause serious disruption. Meanwhile, our scheme does not need to store the user's password or public key with certificate, too.

### 5.3. Mutual Authentication with Session Key Agreement

In the verification phase of the proposed scheme, the service server *S*_*j*_ can authenticate the validity of *U*_*i*_ by checking if *X* = *B*_*i*_′ · *P* − *W*_*i*_′ · *P*_pub_ holds. *U*_*i*_ can verify the public key of *S*_*j*_ Pub_*j*_ = *H*(SID_*j*_||*W*_*j*_) · *P*_pub_ + *W*_*j*_ with *W*_*j*_ to confirm that *S*_*j*_ is the objective service server; meanwhile check the equation *M*_*j*_′ = *M*_*j*_ to affirm that the login message is received by *S*_*j*_. Only when all previous equations are satisfied, the session continues and the communication parties agree on a shared session key SK = *H*(*W*_*i*_||*K*_*ij*_||*T*_*ij*_). For the aforementioned analysis, our scheme can achieve mutual authentication with session key agreement.

### 5.4. No Synchronization Clock

 In our scheme, both the user and the service server employ the random points *R*_*i*_, *R*_*j*_ to interactive with each other. The timestamp does not appear in the proposed scheme; therefore the synchronization clock problem can also be abstained in the session key.

### 5.5. Anonymity

 In the user registration phase, the identity of the remote user can be protected from disclosure by the secure channel between *U*_*i*_ and RC. In the login and authentication phase, *U*_*i*_'s identity is submitted with CID_*i*_ substituting ID_*i*_, nobody can learn the user's real identity, and *S*_*j*_ can only verify the user's validity cannot obtain the real ID_*i*_ with the received message. To general adversary, he/she can extract the smart card and intercept the login message, but he can do nothing to crack the user's identity due to the resistance to collision of the hash function. Therefore, we claim that our scheme can provide the user anonymity.

### 5.6. Security of the Session Key

1. *Perfect Forward Secrecy and Backward Secrecy.* In this scheme, the session key is established by *W*_*i*_, *K*_*ij*_, *T*_*ij*_, where *K*_*ij*_ and *T*_*ij*_ rely on the random values *r*_*i*_ and *r*_*j*_. *r*_*i*_ and *r*_*j*_ are independently generated in each session, are also changed for each authentication phase and are not correlated. The adversary cannot use current session key to derive forward and backward session key. Hence, we claim that our scheme achieves perfect forward secrecy and backward secrecy.
2. *Known Session Key Security.* In this scheme, the session key SK = *H*(*W*_*i*_||*K*_*ij*_||*T*_*ij*_) is composed of *W*_*i*_, *K*_*ij*_ and *T*_*ij*_. Assume that the adversary can seize a session key SK_*mn*_; he cannot obtain the parameters *W*_*m*_, *K*_*mn*_, and *T*_*mn*_ attributed to the one-way hash function *H*(·). Since *K*_*mn*_ and *T*_*mn*_ consist of *R*_*m*_, *R*_*n*_, which are independent for each session, no session keys rely on each other. Furthermore, though the adversary can intercept the current transmitted message *R*_*m*_′, *R*_*n*_′, he cannot compute the new session key SK_*mn*_'s components *K*_*mn*_′ without the server's private key or *T*_*mn*_′ due to the CDH problem's difficulty.
3. *No Key Control.* In this scheme, the session key consists of *W*_*i*_, *K*_*ij*_, *T*_*ij*_, where partial parameters *K*_*ij*_, *T*_*ij*_ are generated by Diffie-Hellman key exchange form; thereby the fairness of the session key can be guaranteed. More specifically, *K*_*ij*_ = *s*_*j*_ · *R*_*i*_ = *r*_*i*_ · Pub_*j*_, *T*_*ij*_ = *r*_*j*_ · *R*_*i*_ = *r*_*i*_ · *R*_*j*_, *R*_*i*_ and *R*_*j*_ are respectively provided by the user and the server; therefore either party is in vain attempting to preselect or control the session key.

### 5.7. Various Common Attacks

 Our proposed remote user authentication scheme for multiserver architecture cannot only meet the previous security features, but also be against various known attacks, such as impersonation attack, and stolen smart card attack. We will discuss the following extra four attacks, the others can refer to [11, 18].

1. *Impersonation Attack. *If an adversary tries to impersonate as a legitimate user to log into the server, he/she must first forge a valid login request message (CID_*i*_, *R*_*i*_). However, the adversary cannot compute a new and legal login message without knowing ID_*i*_ or *B*_*i*_. Suppose that the adversary can steal the smart card of the user *U*_*i*_ by virtue of some approaches, he is still unable to calculate *B*_*i*_ for the reason that he has no information about *A*_*i*_ and ID_*i*_. Moreover, even if the adversary utilizes (CID_*i*_, *R*_*i*_) to log into *S*_*j*_, he cannot pass the verification $X\overset{?}{=}B_{i}^{\prime }\cdot P-W_{i}^{\prime }\cdot P_{\text{pub}}$ because he is unable to provide correct *P*_*ij*_ without *B*_*i*_ or *K*_*ij*_. The adversary cannot obtain the valid session key. Under the situation, our proposed scheme can withstand the impersonation attack.
2. *Stolen Smart Card Attack.* We assume that *U*_*i*_'s smart card is stolen or lost; the adversary picks it and has the ability to breach the information stored in the smart card (*C*_*i*_, *D*_*i*_, *E*_*i*_, *H*(·), *b*_*i*_). Yet on the one hand, it is impossible to guess *A*_*i*_ and ID_*i*_ correctly at the same time, on the other hand, *s* and *x* are, respectively, private key and secret value of RC, so the adversary cannot derive *B*_*i*_. Consequently, the adversary cannot fabricate a valid login message or compute the session key. That is the reason that our proposed protocol is secure against the stolen smart card attack.
3. *Off-Line Password Guessing Attack.* Assume that the adversary guesses a password PW′ from the dictionary; he can compute *A*_*i*_ = *H*(PW′||*b*_*i*_), *B*_*i*_ = *C*_*i*_ ⊕ *H*(*A*_*i*_) but fails to calculate other information without ID_*i*_ or *K*_*ij*_. The adversary cannot examine whether the guessed password PW′ is correct without comparing parameters. Hence, the adversary can extract the smart card information and intercept the transmitted message in public channel, but our proposed scheme can resist the off-line password guessing attack.
4. *Man-in-the-Middle Attack.* When an adversary wants to perform the man-in-the-middle attack, he can intercept the login message, communicate, and share the session key with the server. In the proposed scheme, even if the adversary gets the message in public channel, he cannot calculate *W*_*i*_, *K*_*ij*_, or *T*_*ij*_ without ID_*i*_ or other random values *r*_*i*_, *r*_*j*_. Consequently, our scheme can resist the man-in-the-middle attack.
5. *Server Spoofing Attack.* When a valid but malicious server *S*_*m*_ wants to cheat *U*_*i*_ on behalf of *S*_*j*_ and obtain the session key, he needs to know both the witness and private key of *S*_*j*_. In our scheme, *S*_*m*_ cannot provide the correct witness, and the user *U*_*i*_ cannot pass the server's public key verification. Even if *S*_*m*_ intercepts *W*_*j*_, he cannot check the equation *X* = *B*_*i*_′ · *P* − *W*_*i*_′ · *P*_pub_ since he does not obtain *K*_*ji*_ without knowing the private key *s*_*j*_. Finally, the adversary fails to share the session key with the user *U*_*i*_. Therefore, our scheme can resist the server spoofing attack.

### 5.8. Local Password Verification

In our scheme, *U*_*i*_ can account whether the used smart card matches with himself by checking *C*_*i*_′ = *C*_*i*_ before logging into *S*_*j*_, and thus accomplish the user password verification locally. Through the previous equation, *U*_*i*_ can avoid network resource wasting caused by wrong password. Because until the authentication phase *S*_*j*_ can authenticate user's validity and password appropriateness; in other words, wrong password cannot be detected until the authentication phase. Therefore, our scheme can achieve local password verification.

At last, the functionality comparisons among our and other previously proposed schemes, such as [8, 9, 11, 12, 17, 18], are listed in Table 2. In particular, we can clearly see that the other schemes do not assist in the impersonation attack except our proposed scheme. Thus, it is obvious that our proposed scheme is superior to the others in accordance with all of essential comparative items. In addition, unlike the other related public key-based multiserver authentication schemes [17, 18], ours can achieve the user anonymity and local password verification. On the whole, our proposal is the only one that can satisfy all the functionalities for the multiserver architecture.

### 5.9. Performance

 Under multiserver architecture, the computational cost is a key issue to evaluate whether a remote user authentication scheme is efficient because of mobile devices' constrained resources and computing capability. Before analyzing the computational cost of each phase, define some notations and equivalence relationship first:

- *T*_*e*_: the time to compute a bilinear pairing map;
- *T*_*M*_: the time to compute a point multiplication on the elliptic curve group;
- *T*_*A*_: the time to compute a point addition on the elliptic curve group;
- *T*_*H*_: the time to compute a hash function;
  - *T*_*e*_ = 20*T*_*M*_;
  - *T*_*M*_ = 6*T*_*A*_.

**Figure 2:**
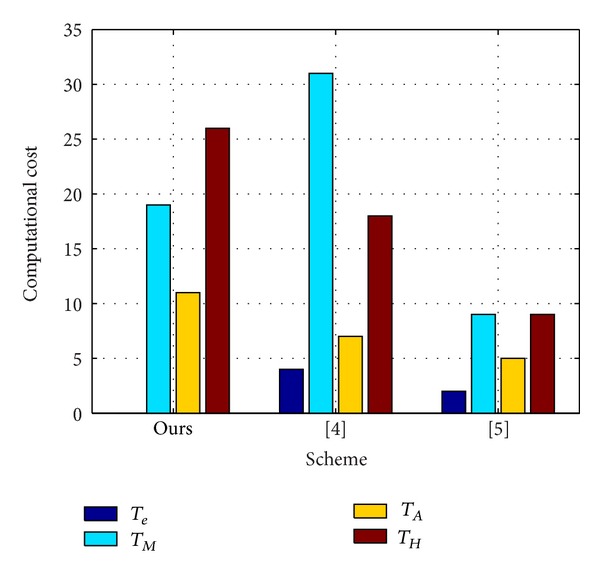
Performance comparison between our scheme and others.

The XOR operation, modular multiplication, and modular addition operation are negligible during evaluating the performance. In the following, we will give the computational cost of five phases individually. In the server registration phase, the computational cost is 5*T*_*M*_ + 4*T*_*A*_ + 2*T*_*H*_. The user registration phase consumes 2*T*_*M*_ +  2*T*_*A*_ + 6*T*_*H*_. When the user logs into the server, it costs *T*_*M*_ + 4*T*_*H*_. During verification of each other between the server and the user, 9*T*_*M*_ + 3*T*_*A*_ + 6*T*_*H*_ is demanded. The computational cost of the password change phase is 2*T*_*M*_ + 2*T*_*A*_ + 8*T*_*H*_. The detailed cost comparisons with the related authentication schemes [17, 18] are illustrated in Table 3. At the same time, we show the implementation result in Figure 2, which can show the computational cost contrast more intuitively. Table 3 and Figure 2 can clearly indicate that our proposal needs no pairing operation, while [18] contains 4*T*_*e*_ and [17] contains 2*T*_*e*_. Because the relative computational cost of a pairing is approximately 20 times higher than that of the point multiplication over elliptic curve group, we can find that the computational cost of ours is obviously much less than that of others by removing pairing operation.

From Tables 2 and 3, we can make a conclusion that our remote authentication scheme has more security features and lower computational cost among the existing related works, which satisfies the requirements for the multiserver architecture.

## 6. Conclusions

 An anonymous and efficient remote user authentication scheme for the multiserver architecture is proposed in this paper and the self-certified public keys are employed. Our scheme can satisfy all of the requirements needed for achieving secure authentication in multiserver environments, as compared with the previously proposed schemes. Moreover, the proposal succeeds to both achieve the user's identity anonymity and remove the pairing operation, which makes that the proposed scheme can provide more advantages and be more practical for the actual applications. Additionally, we analyze the security and performance of our proposal and make comparisons with other related works. From these analysis and comparisons, we can reach a conclusion that our proposed scheme owns more functionalities and attains higher efficiency.

## Figures and Tables

**Table 1:** Notations used in proposed scheme.

Notations	Descriptions
RC	The registration center
*S*_*j*_	The *j*th service server
*U*_*i*_	The *i*th user with mobile device
*s*	The private key of RC
SID_*j*_	The identity of *S*_*j*_
ID_*i*_	The identity of *U*_*i*_
*P*	A generator of group *G*
*H*(·)	A one-way hash function
PW_*i*_	The password of *U*_*i*_
SK	A session key shared between *U*_*i*_ and *S*_*j*_
*x*	The secret value maintained by RC
⊕	A simple Exclusive-OR operation
||	The concatenation operation

**Table 2:** Functionality and security comparison with the related schemes.

Functionality	Ours	[18]	[17]	[12]	[11]	[9]	[8]
No repetitive registration	Y	Y	Y	Y	Y	Y	Y
No verification table	Y	Y	Y	Y	Y	Y	Y
Mutual authentication with key agreement	Y	Y	N	N	Y	N	N
No synchronization clock	Y	Y	N	Y	Y	N	Y
Change password freely	Y	Y	Y	Y	Y	Y	Y
Anonymity	Y	N	N	Y	Y	Y	Y
Perfect forward and backward secrecy	Y	Y	N	N	Y	N	N
No key control	Y	Y	Y	Y	Y	Y	Y
Known session key security	Y	Y	Y	Y	Y	Y	Y
Impersonation attack	Y	N	N	N	N	N	N
Stolen smart card attack	Y	Y	N	N	N	N	N
Off-line password guessing attack	Y	N	N	Y	N	N	N
Man-in-the-middle attack	Y	Y	N	Y	Y	N	N
Server spoofing attack	Y	N	N	Y	Y	Y	N
Local password verification	Y	N	N	Y	Y	Y	Y

**Table 3:** Cost comparison with the related schemes.

Phase	Ours	[18]	[17]
Server registration	5*T*_*M*_ + 4*T*_*A*_ + 2*T*_*H*_	5*T*_*M*_ + 4*T*_*A*_ + 2*T*_*H*_	—
User registration	2*T*_*M*_ + 2*T*_*A*_ + 6*T*_*H*_	3*T*_*M*_ + 2*T*_*H*_	3*T*_*M*_ + 2*T*_*H*_
Login	*T*_*M*_ + 4*T*_*H*_	3*T*_*M*_ + *T*_*A*_ + 3*T*_*H*_	3*T*_*M*_ + 2*T*_*H*_
Verification	9*T*_*M*_ + 3*T*_*A*_ + 6*T*_*H*_	2*T*_*e*_ + 8*T*_*M*_ + 2*T*_*A*_ + 5*T*_*H*_	2*T*_*e*_ + *T*_*M*_ + *T*_*A*_ + 2*T*_*H*_
Password change	2*T*_*M*_ + 2*T*_*A*_ + 8*T*_*H*_	2*T*_*e*_ + 15*T*_*M*_ + 6*T*_*H*_	2*T*_*M*_ + *T*_*H*_

Total	19*T*_*M*_ + 11*T*_*A*_ + 26*T*_*H*_	4*T*_*e*_ + 31*T*_*M*_ + 7*T*_*A*_ + 18*T*_*H*_	2*T*_*e*_ + 9*T*_*M*_ + 5*T*_*A*_ + 9*T*_*H*_
